# Supplementary material for: Enhanced cadmium binding ability in response to novel modifications in a Paramecium cadmium metallothionein PMCd1
Source: PLoS One. 2025 May 22;20(5):e0324322. doi: 10.1371/journal.pone.0324322 (PMC12097635; doi:10.1371/journal.pone.0324322)
Supplement: Attachment — Submitted filename: General Comments for PLOS.docx [file pone.0324322.s002.docx]

**General Comments:**

The study investigates the potential to enhance cadmium binding in a metallothionein (PMCd1) by introducing additional cysteine residues, an approach that is of significant interest in the field of metal detoxification. The findings are promising, particularly the demonstration that the S20C mutation results in a 9.1-fold increase in metal uptake. However, the novelty of this work could be more clearly articulated, especially regarding how the specific placement of cysteine residues enhances cadmium binding compared to existing studies on metallothioneins. The impact of the findings on environmental or biotechnological applications, such as bioremediation, should be more fully explored.

1. A brief discussion of why these sites cysteine residues (S20C, R180C, and Y185C) were targeted and how they relate to cadmium binding in the native protein structure would help clarify the objectives of the study.

2. While the study clearly states that the goal is to enhance metal binding capacity, the hypothesis for why introducing cysteine residues at these specific positions will improve binding should be more thoroughly explained. Are these positions related to known binding sites, or is the enhancement due to structural changes in the protein?

**Specific Comments:**

**Introduction**:

1. The introduction should provide a more detailed background on the role of metallothioneins in metal binding and detoxification, particularly in protozoan ciliates like *Paramecium*. While the significance of metallothioneins in metal homeostasis is mentioned, it would benefit from a more specific discussion of how metallothioneins from ciliates compare to those from other organisms, particularly in their metal uptake abilities.

2. Please explain why *Paramecium* was chosen as the model organism for this study. How does it compare with other organisms used in similar studies, and why is its cadmium detoxification system particularly relevant for this type of research?

3. In the material and methods section how were the specific mutations (S20C, R180C, Y185C) chosen, and what strategy was used to confirm their introduction into the PMCd1 gene?

4. It would be helpful to include more details on the expression conditions in E. coli BL21 cells. Were any specific conditions used to enhance protein expression or stability? Additionally, were the proteins purified for further analysis, or was the activity assessed directly in the bacterial lysates?

5. The metal uptake assay is a crucial experiment. Please provide more details on the specific methodology used to measure cadmium uptake. For example, were cadmium levels measured using atomic absorption spectroscopy (AAS) or another method? How were the results normalized (e.g., per cell count or protein concentration)?

**Results**:

The role of the individual cysteine residues (S20C, R180C, Y185C) should be further analyzed. Why does the S20C mutation show the highest increase in metal uptake? Are there any structural or functional reasons behind this observation?

**Discussion**:

1. The discussion should provide a more in-depth analysis of the mechanisms underlying the enhanced metal binding. For example, how do the introduced cysteine residues affect the protein structure and cadmium binding sites? Do these mutations affect the coordination of cadmium ions or alter the protein’s stability in a way that facilitates greater binding?

2. The implications of these findings for *Paramecium*’s ability to deal with cadmium contamination should be explored in more detail. How might the enhanced cadmium binding ability contribute to the organism’s resistance to cadmium toxicity, and what are the potential applications of this in environmental cleanup or bioremediation?

**Conclusion**:

The conclusion should summarize the key findings, emphasizing the practical implications of the enhanced cadmium binding ability. It would be valuable to discuss potential next steps for the research, such as further optimizing the protein for use in cadmium removal from contaminated environments or exploring the stability and effectiveness of the mutant in real-world conditions.

**Minor Comments:**

1. The manuscript is generally well-written but could benefit from more concise phrasing in certain sections. For example, some sentences in the introduction are lengthy and could be restructured for clarity.
2. Ensure that technical terms, such as "site-directed mutagenesis," are consistently defined and used appropriately throughout the manuscript.
3. The reference list should be expanded to include more recent studies on the engineering of metallothioneins for metal binding enhancement, particularly those focusing on cadmium. Also, ensure that all references are properly formatted according to the journal's style.
4. It may be beneficial to include additional data, such as stability assays or metal binding kinetics, to further support the conclusions drawn from the metal uptake experiments.

**Overall Recommendation:**

The manuscript presents a valuable contribution to the field of metallothionein engineering for cadmium removal, with promising results for the S20C mutant of PMCd1. However, additional details in the methods, more comprehensive data presentation, and a deeper discussion of the molecular mechanisms underlying the enhanced metal binding are needed to fully strengthen the manuscript. With these revisions, the manuscript has the potential for publication.
