# Supplementary material for: Enhanced cadmium binding ability in response to novel modifications in a Paramecium cadmium metallothionein PMCd1
Source: PLoS One. 2025 May 22;20(5):e0324322. doi: 10.1371/journal.pone.0324322 (PMC12097635; doi:10.1371/journal.pone.0324322)
Supplement: Attachment — Submitted filename: Rebuttal-Response to reviewers.docx [file pone.0324322.s004.docx]

**REBUTTAL – Response to Reviewers**

**Academic Editors Comments**

**Key Comments**

1. The novelty of the study should be clearly articulated in the introduction section. This is crucial to highlight the unique contributions of this work.

**Major part of Introduction has been rewritten, with an aim of highlighting uniqueness of the protein under investigation. The arguments and explanations have been added in lines 54-96 of the revised version.**

2. The quality of the figures is inadequate, particularly Figures 1 and 6. The authors are requested to provide high resolution images to ensure clarity and proper visualization.

**The resolution of all figures (including Figures 1 and 6)	has been improved.**

**Reviewer’s Comments**

**General Comments:**

The study investigates the potential to enhance cadmium binding in a metallothionein (PMCd1) by introducing additional cysteine residues, an approach that is of significant interest in the field of metal detoxification. The findings are promising, particularly the demonstration that the S20C mutation results in a 9.1-fold increase in metal uptake. However, the novelty of this work could be more clearly articulated, especially regarding how the specific placement of cysteine residues enhances cadmium binding compared to existing studies on metallothioneins. The impact of the findings on environmental or biotechnological applications, such as bioremediation, should be more fully explored.

(i) **Novelty of the work has been explained in three paragraphs – Lines 239-248, 252-255, 306-320 under “Discussion”.**
(ii) **Future implications of this study have been described in Lines 322-330. ”**

1. A brief discussion of why these sites cysteine residues (S20C, R180C, and Y185C) were targeted and how they relate to cadmium binding in the native protein structure would help clarify the objectives of the study.

**The proposed metallothionein (203 aa) has 17 CX_2_C motifs arranged in six repeats X_m_CX_2_CX_3_CX_2_CX_n_CX_2_C (m=8-15, n=5-10); each repeat having three CX_2_C motifs with the exception of the sixth repeat with two motifs only. Keeping in view the above deduced pattern of amino acids in the native metallothionein proteins in ciliates, three cadmium binding pockets were identified and these mutations (S20C, R180C, and Y185C)** **were done in pocket 1, 2 and 3, respectively. This decision was based on the fact that thiol group of Cys has high affinity for metal ions. None of these mutations had any effect on the 3D structure of PMCd 1 (Fig. 2).**

**The procedure adopted for the above site directed mutagenesis , has been explained in a new paragraph added under Materials & Methods. See Lines 333-397.**

2. While the study clearly states that the goal is to enhance metal binding capacity, the hypothesis for why introducing cysteine residues at these specific positions will improve binding should be more thoroughly explained. Are these positions related to known binding sites, or is the enhancement due to structural changes in the protein?

**As explained above, the rationale for selecting specific sites for introducing cysteins was based on the unique CX_2_C motifs in these proteins and the metal binding capacity of the thiol groups in cysteins. Since the three D structures of proteins remained unchanged after mutations, the enhanced cadmium binding capacity can be safely attributed to increased number of cysteine residue in their specific environments and not due to any structural change.**

**Specific Comments:**

**Introduction**:

1. The introduction should provide a more detailed background on the role of metallothioneins in metal binding and detoxification, particularly in protozoan ciliates like *Paramecium*. While the significance of metallothioneins in metal homeostasis is mentioned, it would benefit from a more specific discussion of how metallothioneins from ciliates compare to those from other organisms, particularly in their metal uptake abilities.

**Metallothioneins of ciliates are unique proteins in terms of their high cysteine content, which exhibits a strictly conserved modular-submodular structure. Compared to MTs from other organisms, ciliate MTs are longer, have high cysteine content and can bind a wide range of metal ions including Cd, Zn, CU , which is because of their high cysteine content and unique structural features. The gene expression of ciliate MTs show multistress and differential response to various metals and environmental stressors.**

**Ciliate MTs sequester toxic metal ions preventing them from interaction with cellular compounds and causing damage. They also provide protection against oxidative stress induced by metal ions. By binding and neutralizing metal ions, they help maintain cellular redox balance.**

**The above information has been added in lines 305-320 under Discussion, as well as in Material and Methods in lines 334-379.**

**The required information has also been added in Introduction.**

2. Please explain why *Paramecium* was chosen as the model organism for this study. How does it compare with other organisms used in similar studies, and why is its cadmium detoxification system particularly relevant for this type of research?

**The absence of a cell wall in the vegetative stage of these eukaryotic microorganisms offers a higher sensitivity to environmental pollutants compared to yeasts and bacteria, and hence evince a faster cellular response. Most of the previous work reported from other laboratories on metallothionein in ciliates has been done on *Tetrahymena.* For this study, *Paramecium* spp. were selected for their convenient availability in the local environment, and relatively large size (40–50 lm), making their manipulations easier. They grow rapidly giving a high cell density (approximately 10^6^ cells/ml) in a variety of media and different culture conditions. Moreover the comparison of metallothionein proteins of these two different species of ciliates were expected to provide interesting comparison and new lines of investigation regarding evolutionary development of these microorganisms.**

**This information has been added in lines 238-248 in the revised version.**

3. In the material and methods section how were the specific mutations (S20C, R180C, Y185C) chosen, and what strategy was used to confirm their introduction into the PMCd1 gene?

**Firstly, 3D structure of the protein was predicted through SWISS_MODEL and AlphaFold. Possible Cd^2+^ binding sites in the proposed model were predicted through MIB2 (metal ion-binding) tool which is both a metal ion binding site prediction and docking server. The sites with docking score >1.5 were selected. Amongst these, sites present in close proximity were merged together to form five potential cadmium binding pockets. The mutations were proposed in pocket regions keeping in mind that none of these affected the 3D structure of PMCd1. Amongst these, R180C mutation resulted in the same pattern of** **X_m_CX_2_CX_3_CX_2_CX_n_CX_2_C as in rest of the protein. Multiple alignment of PMCd1 with the homologous sequences in other *Paramecium* spp. revealed that these contain Cys at position 180 as shown in the following figure. In the figure *Paramecium* sp. Represents PMCd1 while rest are homologouse sequences of hypothetical proteins of other species including CAD8205572.1 [*Paramecium pentaurelia*], XP_001428442.1 [*P. tetraurelia*], CAD8199496.1 [P. octaurelia] and CAD8116148.1 [*P. sonneborni*].**

```
Paramecium sp.     MDKVNNNSCTVCPTLCATCSDANNCTSCDIGYYLDKTNPSAVTCTKCNNPCYGCVDNATK
P.pentaurelia      GYYVNNNSCTVCPTQCAKCSDANNCTSCDIGFFLDSTNPSAVTCTKCTNPCYGCVDNATK
P.tetraurelia      YYVNGSKVCTLCAAQCAKCSDANNCTGCDIGFFLDKTNPNAVTCTKCTNPCYGCVDNATK
P.octaurelia       YYVNGSNACTVCAAQCAKCSDANNCTGCDIGFFLDSTNPNAVTCTKCTNPCYGCVDNATK
P.sonneborni       GYYANNKTCTICPNQCADCSDANNCTACNKGYFLDKTNPNAVTCSNCNNPCYGCFDNATK
                       ..: **:*.  ** ********.*: *::**.***.****::*.******.*****

Paramecium sp.     CTACDQGLVLDSVNHTCNQCSPECTSCDQADPKNCQTCANGYYYNDNNQCKQCSNLCKTC
P.pentaurelia      CNACDSGLVLDNVNHTCNQCSPECTSCNSADPKNCQTCANGYYYNNNNQCLQCSNLCKTC
P.tetraurelia      CTVCDSGLVLDDVNHTCNQCSPECTSCSQSDPKNCQSCSPGYYYNNNNQCLQCSNLCKTC
P.octaurelia       CTVCDSGLVLDSVNHTCNQCSPQCTSCSQSDPTNCQSCSPGYYYNNNNQCLQCSNLCKTC
P.sonneborni       CSDCDNGLVLDEVNHTCKQCSPECTTCLQNDPKNCQTCADGYYYNNNNQCLQCSNLCKTC
                   *. **.*****.*****:****:**:* . **.***:*: *****:**** *********

Paramecium sp.     QDQNGKGENYCTSCFSGFYQPTGQNTCKICKQPCKTCET---AEDHCLTCYDGNFWD--S
P.pentaurelia      SNNN---KDYCTSCLSGFYQPTGQNTCKVCTEPCKTCEN---TGDHCLSCYSGNFWD--S
P.tetraurelia      SQND---KNYCTACYDGFYQPTGQNTCKVCIEPCKTCKNS-TSGDQCLSCYSGDFWD--D
P.octaurelia       SNND---KNYCTSCYAGFYQPTGQNTCKVCIEPCKTCKDS-TSGDQCLSCYSGDFWD--E
P.sonneborni       DQNN---KDFCTSCYDGFYQPNGQNTCKVCKQPCLTCKSQQAEGDNCLTCFKGDYLDNST
                   .:::   :::**:*  *****.******:* :** **:      *:**:*:.*:: *

Paramecium sp.     TNFLRKQCQYPCVFCNDLTTCDTCECCK
P.pentaurelia      TNFLCKQCQYPCVNCNDLTTCVTCEKGY
P.tetraurelia      TNFLCKQCQYPCVSCTNLTTCKTCEKGY
P.octaurelia       PNSLCKQCQYPCVSCTNLTTCVTCEKGY
P.sonneborni       TPPSCVQCVYPCVECQDENTCKSCQKGY
                   .     ** **** * : .** :*:
```

**Y185C** mutation resulted in the generation of a CXCXC, a known motif of *Tetrahymena* MT (Gutiérrez et al , 2011). **S20C** mutation was randomly selected to generate CXXCC.

The introduction of each mutation was confirmed through sequencing of each of the recombinant vectors (pUC57-PMCd1, pUC57-S20C, pUC57-R180C and pUC57-Y185C).

4. It would be helpful to include more details on the expression conditions in E. coli BL21 cells. Were any specific conditions used to enhance protein expression or stability? Additionally, were the proteins purified for further analysis, or was the activity assessed directly in the bacterial lysates?

**All details are already provided in lines 434-440 and then 443-474. No specific condition was used to enhance protein expression or stability, except for induction with IPTG, which has been mentioned in lines 448-451 as well as 468-470. Proteins were not further purified nor any activity was determined in the cell lysates.**

5. The metal uptake assay is a crucial experiment. Please provide more details on the specific methodology used to measure cadmium uptake. For example, were cadmium levels measured using atomic absorption spectroscopy (AAS) or another method? How were the results normalized (e.g., per cell count or protein concentration)?

**Yes, the cadmium levels were determined through atomic absorption spectroscopy (already mentioned in line 455). Details are provided in lines 443-474. The results were normalized per dry weight of cell pellet.**

**Results**:

The role of the individual cysteine residues (S20C, R180C, Y185C) should be further analyzed. Why does the S20C mutation show the highest increase in metal uptake? Are there any structural or functional reasons behind this observation?

**Introduction of cysteine at three specific sites having peculiar motifs such as X_m_CX_2_CX_3_CX_2_CX_n_CX_2_C was aimed at increasing the cadmium uptake by the metallothionein protein. The purpose was to increase number of thiol groups which have great affinity for proteins. Cysteines act as ligand which coordinate with metal ions to form more stable and active protein conformation. These proteins have specialized domains that facilitate the uptake and transport of metals, ensuring efficient metal homeostasis within the cell. S20C mutation gave the highest increase in metal uptake probably because of the specific amino acid environments of the protein in this region.**

**Discussion**:

1. The discussion should provide a more in-depth analysis of the mechanisms underlying the enhanced metal binding. For example, how do the introduced cysteine residues affect the protein structure and cadmium binding sites? Do these mutations affect the coordination of cadmium ions or alter the protein’s stability in a way that facilitates greater binding?

**The metal binding capacity of a protein can be enhanced either by introducing more cysteine residues in the protein by site directed mutagenesis or modifying the structure of metallothionein to create more favourable binding sites for metal ions. In this study we achieved the objectives by site directed mutagenesis.**

**The enhanced cadmium binding contributes to organism’s resistance to cadmium toxicity. This resistance is attributable to (1) production of metallothionein and phytochelatins by the organism which bind Cd ions, sequestering them into vacuoles and thus prevent them from interacting with cellular compounds (2) transportation of Cd ions out of cells, reducing the intracellular Cd concentration and mitigating its toxic effect. (3) enzymatic detoxification of cadmium by reducing the availability of free cadmium ions, thereby decreasing ROS production and mitigating oxidation stress. Some enzymes can detoxify Cd by converting it into less harmful forms. Fort example glutathione reductase and other antioxidant enzymes can neutralize the oxidative stress caused by Cd (4) development of structural and genetic adaptations that enhance their ability to bind and detoxify cadmium. For example several functional groups (such as amines, carboxyl, phosphate and hydroxyl) on the bacterial cell wall can bind with cadmium ions , thus preventing their entry into the cell and (5) enhancement of the expression of genes involved in cadmium binding and detoxification such as Signal molecules like phytohormones and ROS activating pathways. Transcription factors such as WRKY, MYB, bZIP play a key role in regulating these responses**

**The above has been added in Discussion (Lines 306-320).**

2. The implications of these findings for *Paramecium*’s ability to deal with cadmium contamination should be explored in more detail. How might the enhanced cadmium binding ability contribute to the organism’s resistance to cadmium toxicity, and what are the potential applications of this in environmental cleanup or bioremediation?

**Paramecium metallothionein with enhanced cadmium binding capability can serve as bioindicator for cadmium pollution in aquatic environment. Its sensitivity to cadmium makes it a useful organism for monitoring and assessing the level of cadmium contamination in water bodies. Its implications fall within the domain of bioremediation: By harnessing their natural ability to sequester and detoxify cadmium, effective methods to reduce cadmium levels in polluted water bodies and soil can be developed. These methods will incur less financial investment for a more sustainable and long term solution. This technology is likely to have lower environmental impact compared to chemical or physical methods.**

**The above has been added in lines 323-330.**

**Conclusion**:

The conclusion should summarize the key findings, emphasizing the practical implications of the enhanced cadmium binding ability. It would be valuable to discuss potential next steps for the research, such as further optimizing the protein for use in cadmium removal from contaminated environments or exploring the stability and effectiveness of the mutant in real-world conditions.

**Conclusion has been modified in the light of suggestions (see lines 507-516). Due to their metal binding properties, metallothioneins have the potential applications in bioremediation, medicine and biotechnology. They can be used to develop biosensors for detecting metal ions and create therapeutic agents for treating metal related diseases.**

**Minor Comments:**

1. The manuscript is generally well-written but could benefit from more concise phrasing in certain sections. For example, some sentences in the introduction are lengthy and could be restructured for clarity.

**Done.**

2. Ensure that technical terms, such as "site-directed mutagenesis," are consistently defined and used appropriately throughout the manuscript.

**Done**

3. The reference list should be expanded to include more recent studies on the engineering of metallothioneins for metal binding enhancement, particularly those focusing on cadmium. Also, ensure that all references are properly formatted according to the journal's style.

**There are few studies that report enhanced bioremediation potential through genetically modified microbes or plants. For example, Sauge-Merle  et al. (2003) showed that bacterial cells expressing *Arabidopsis thaliana* phytochelatin exhibited enhanced toxic metal accumulation. In all similar studies, the organisms are genetically engineered through introduction of metallotionien genes and their subsequent expression. To best of our knowledge, there is no report about modification of a metallothionien through Cys additions. This is the first report and thus can not be compared with anyone.**

**Sauge-Merle SCuiné S, Carrier P, Lecomte-Pradines C, Luu D, Peltier G. 2003. Enhanced toxic metal accumulation in engineered bacterial cells expressing *Arabidopsis thaliana* Phytochelatin Synthase. Appl Environ Microbiol  69:.<https://doi.org/10.1128/AEM.69.1.490-494.2003>**

4. It may be beneficial to include additional data, such as stability assays or metal binding kinetics, to further support the conclusions drawn from the metal uptake experiments.

**It is true that stability assays and metal binding kinetics are crucial for understanding the behaviour of metal ions in biological systems and their interaction with proteins. Assessment of thermal stability of proteins, their exposure to denaturants at a constant temperature, and changes in fluorescence after the protein unfolds or refolds in the presence of metals ions provides valuable insight into the stability of protein-metal interaction. In addition, as the honorable reviewer has pointed out, metal binding kinetics such as association-disassociation kinetics, Kd values , competitive binding of metals other than cadmium with proteins could also further augment the results of this study. Since the scope initially set for this study did not involve the stability and metal binding kinetics, this part of the study, though important and interesting has not been included in the present manuscript. I am however thinking of incorporating this aspect in our future plan of work on metallothioneins.**

**Overall Recommendation:**

The manuscript presents a valuable contribution to the field of metallothionein engineering for cadmium removal, with promising results for the S20C mutant of PMCd1. However, additional details in the methods, more comprehensive data presentation, and a deeper discussion of the molecular mechanisms underlying the enhanced metal binding are needed to fully strengthen the manuscript. With these revisions, the manuscript has the potential for publication.

**Reviewer #2:**

The manuscript is well written and comprehensive in it's approach but needs further modification for it's consideration for publication in the journal.
Authors, kindly, go through the following comments for needed correction:
1. Revise the discussion section as it appears more of a summary of the work done.

**Done. See Lines 238-330.**

2. Expand the conclusion section.

**Done. See Lines 507-516.**

3. Go through the manuscript for any grammatical error or abbreviations. Use same abbreviations throughout the manuscript, eg, hour is mentioned as h or hs in some sections while hr or hour in figures. Also the font size is varying in Bioinformatics analysis for docking. Kindly correct it.

**Thank you for pointing it out. For the sake of uniformity we replaced hour, hs and hr with h throughout the text.**

4. Mention the versions of the docking softwares used and if possible mention the links of these softwares and sites for accessibility of readers.

**AutoDock Vina 1.2.x. was used to predict binding modes of ligands to their target protein. All source code is available under the Apache License version 2.0 from github.com/ccsb-scripps/AiutoDock Vina	and the Python package index	Pypi.org/project/vina.**

**This information has also been added in lines 486-488.**

5. Explain the rational behind retention of charge on various ions and similar binding ability of metal ions with similar ionic radii as that of cadmium in the discussion section.

**Retention of charges on ions is primarily because of electrostatic forces. Positive and negative charges attract each other, which then help maintain the charges on ions. Moreover the charge on anion is determined by its electron configuration. Atoms gain or lose electrons to achieve stable electron configuration, often resembling the nearest noble gas. This process results in the formation of ions with specific charges. Moreover metal ions with similar ionic radii often have similar charge densities (ratio of the charge of ions to its volume) , which influence their binding interaction. Ions with similar charge densities can interact similarly with ligands or binding sites. Metal ions with similar ionic radii and charges have similar lattice energies, contributing to their similar binding abilities.**

6. Significant difference among variables is missing in all the graphs except for Fig 7 (a). Include it in all.

**Significant values have been indicated wherever needed.**
